# The Spectrum of Antibiotic Prescribing During COVID-19 Pandemic: A Systematic Literature Review

**DOI:** 10.1089/mdr.2020.0619

**Published:** 2021-12-06

**Authors:** Sara H. Al-Hadidi, Hashim Alhussain, Hamad Abdel Hadi, Alreem Johar, Hadi M. Yassine, Asmaa A. Al Thani, Nahla O. Eltai

**Affiliations:** ^1^ Biomedical Research Center, Qatar University, Doha, Qatar.; ^2^ Infectious Disease Division, Communicable Diseases Centre, Hamad Medical Corporation, Doha, Qatar.; ^3^ Barzan Holdings, Doha, Qatar.

**Keywords:** COVID-19, antibiotics, antimicrobial stewardship, resistance, AMR

## Abstract

***Objectives:*** Over the last decades, there has been a significant increase in antimicrobial prescribing and consumption associated with the development of patients' adverse events and antimicrobial resistance (AMR) to the point of becoming a global priority. This study aims at evaluating antibiotic prescribing during COVID-19 pandemic from November 2019 to December 2020.

***Materials and Methods:*** A systematic review was conducted primarily through the NCBI database, using PRISMA guidelines to identify relevant literature for the period between November 1, 2019 and December 19, 2020, using the keywords: COVID-19 OR SARS-Cov-2 AND antibiotics restricted to the English language excluding nonclinical articles. Five hundred twenty-seven titles were identified; all articles fulfilling the study criteria were included, 133 through the NCBI, and 8 through Google Scholar with a combined total of 141 studies. The patient's spectrum included all ages from neonates to elderly with all associated comorbidities, including immune suppression.

***Results:*** Of 28,093 patients included in the combined studies, 58.7% received antibiotics (16,490/28,093), ranging from 1.3% to 100% coverage. Antibiotics coverage was less in children (57%) than in adults with comorbidities (75%). Broad-spectrum antibiotics were prescribed presumptively without pathogen identifications, which might contribute to adverse outcomes.

***Conclusions:*** During the COVID-19 pandemic, there has been a significant and wide range of antibiotic prescribing in patients affected by the disease, particularly in adults with underlying comorbidities, despite the paucity of evidence of associated bacterial infections. The current practice might increase patients' immediate and long-term risks of adverse events, susceptibility to secondary infections as well as aggravating AMR.

## Introduction

The discovery of antibiotics in the middle of the 20th century was a significant breakthrough for humanity saving millions of lives and preventing significant morbidity and mortality associated with infectious diseases.^1^ A decade after the historical discovery, a noticeable antimicrobial resistance (AMR) was observed escalating to an alarming scale over recent years.^2^ It has been estimated that about 700,000 annual global mortality is attributed to AMR, which attracted the attention of world leaders and international organizations such as the World Health Organization (WHO) all advocating regional and global initiatives to contain the problem.^3^ Antimicrobial Stewardship Programs (ASPs) have been implemented in many health care settings worldwide to curtail inappropriate and excessive antibiotic prescribing, particularly for broad-spectrum antibiotics.^4^ At the end of 2019, the world witnessed a worrying herald of a global pandemic caused by a novel coronavirus coined SAR-CoV-2 leading to the clinical syndrome of COVID-19 disease.^5^ Although the disease causes a respiratory illness primarily, it was noticed from the beginning it is associated with significant secondary presentations, including multisystem complications in need of critical care, particularly for server disease. Since there was no available effective management, antibiotics were frequently prescribed for various rationales with the potential of contributing to AMR.^6^ Although COVID-19 principally is a viral infection not usually responding to antibiotics, it is capable of causing an acute respiratory disease indistinguishable from bacterial infections and creating an environment and complications favoring secondary bacterial infections.^7^ For such reasons, health care professionals were confounded to prescribe antibiotics to treat potential bacterial infections or secondary complications. To comprehend the scale of the problem, a study conducted by the WHO demonstrated that 72% of COVID-19 patients received antibiotics. Nevertheless, only 8% had evidence of documented superimposed bacterial infections.^8^

To add to the complexity of the situation, unverified research at the start of the pandemic advocated combined management with chloroquine/hydroxychloroquine together with the macrolide antibiotic azithromycin led to hasty inclusion in many COVID-19 management guidelines across the globe before establishing better-evaluated efficacy.^9^ Even for patients who warrant treatment during the pandemic, Getahun *et al.*^8^ indicated that antimicrobials were overprescribed for patients admitted to intensive care units (ICUs) in 88 countries where 70% of patients received antibiotics. However, only 54% of patients had suspected or proven bacterial infections. Because of the gravity of the situation, confusion of the optimal management approaches for the novel disease together with the stretching of physical limits and capabilities of health care ASPs; the COVID-19 pandemic created an environment for inappropriate and excessive antibiotic prescribing, which might worsen future AMR through selective pressures. The presented literature review is conducted to examine and highlight the spectrum of antimicrobial prescribing during the COVID-19 pandemic to raise awareness toward potential consequences.

## Materials and Methods

**FIG. 1.**
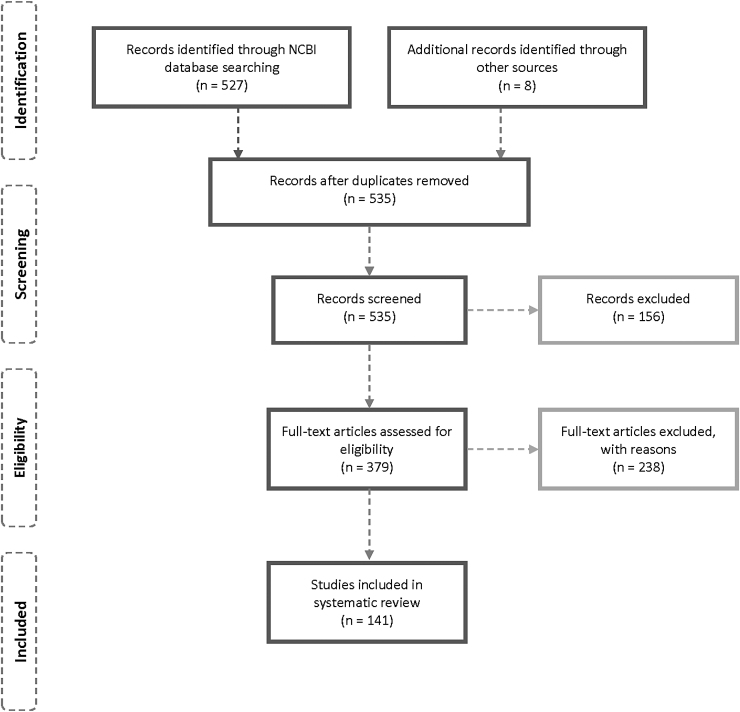
Schematic selection process of included studies.

A literature search was conducted using the PRISMA guidelines for systematic reviews.^172^ The NCBI database was identified as a primary source of related literature because of clinical relevance between November 2019 and December 19, 2020. Adopted search keywords were COVID-19 OR Sars-Cov-2 AND antibiotics restricted to the English language. The search initially resulted in 527 identified titles eventually limited to 133 following applying restrictive criteria. An additional 8 articles were included following searching Google Scholar search engine, bringing the total number to 141 studies. As per the study protocol, only articles covering clinical settings were included, articles limited to basic science, solely microbiological characteristics, experiments, surveys, guidelines, and hypotheses. Those not providing details of antibiotic prescribing were excluded (*n* = 386) (Fig. 1). The information extracted from the included articles comprises types of antibiotics prescribed for COVID-19 patients and the number of those patients, bacterial coinfection, and relevant patient demographic data (age, gender, and country). In addition, if the COVID-19 patient is suffering from any other complications such as hypertension, cardiac disease, diabetes, pregnancy, cancer, and human immunodeficiency virus (HIV) were reported.

## Results

One hundred forty-one articles were included in this review from 28 different countries. The majority of them are from countries worst affected by the pandemic: China (*n* = 55), followed by the USA (*n* = 18), Italy (*n* = 10), UK (*n* = 5), Spain (*n* = 5), Brazil (*n* = 4), Iran (*n* = 4), and India (*n* = 3). Two articles were incorporated from Belgium, Germany, Japan, South Korea, Netherlands, and Saudi Arabia and one from Bhutan, Colombia, France, Ireland, Morocco, Niger, Oman, Philippines, Qatar, Singapore, Switzerland, Taiwan, and Uganda. Fourteen articles were included with no identified country (Table 1).

**Table 1.** Showing Affected Countries, the Total Number of Patients, Number and Percentage of Patients Prescribed Antibiotics, Gender, Age, Prescribed Antibiotics, and Comorbidities

Country	Total number of patients	Number of patients prescribed antibiotic therapy,* n *(%)	Gender	Age, mean ± SD (range)	Prescribed antibiotics and the number of patients prescribed	Comorbidities and the number of patients
South Korea^10^	7,339	2,820 (38.1)	2,970 Male 4,369 Female	47.1 ± 19.0	Unspecified antibiotic: 3,174 Penicillin: 646 Cephalosporins: 1,649 Sulfamethoxazole/trimethoprim: 43 Tetracycline: 33	HT: 1,373 Tuberculosis: 28 COPD: 81 Pneumonia: 513 Asthma: 387 DM: 857 CKD: 48 CLD: 645 CVDs: 455 Cancer: 162 HIV: 4
USA^11^	5,853	4,130 (71)	NA	NA	Doxycycline, azithromycin, levofloxacin, ciprofloxacin, ceftriaxone, and cefepime	Not reported
China^12^	3,309	2,127 (64.28)	1,642 Male 1,667 Female	62 (median)	Unspecified antibiotics	HT: 988 DM: 464 CVD: 242 Cerebrovascular disease: 130 Cancer: 93 CKD: 57 COPD: 42
China^13^	1,123	792 (70.5)	560 Male 563 Female	61 (median)	Azithromycin: 63 Fluoroquinolones: 666 Levofloxacin: 77 Moxifloxacin: 690 Cephalosporins: 220 Penicillin: 50 Carbapenems: 108 Meropenem: 77	HT: 361 Coronary heart disease: 95 Other heart diseases: 46 DM: 147 Cancer: 40 COPD: 40
China^14^	1,099	637 (58)	640 Male 459 Female	47 (median)	Unspecified antibiotics	Not reported
China^15^	970	505 (52.1)	561 Male 409 Female	45.1 ± 17.3	Teicoplanin	Not reported
Netherlands^16^	925	669 (72.3)	583 Male 324 Female	70 (median)	Cefuroxime, amoxicillin, ciprofloxacin	Not reported
China^17^	476	319 (67)	319 Male 205 Female	53 (median)	Unspecified antibiotics	Not reported
China^15^	468	264/330 (80.0)	282 Male 282 Female	53.1 ± 27.6	Teicoplanin	Not reported
China^18^	465	218 (46.88)	243 Male 222 Female	45 (5–88)	Cephalosporins, quinolones, carbapenem, tigecycline, and linezolid	HT: 82 DM: 28 CLD: 19 Cancer: 5 (1.08%) CKD: 5 Heart disease: 3 Pediatric: 3 Pregnancy: 2
China^19^	450	225 (50)	228 Male 222 Female	46.2 ± 15.1	Quinolones: 190 Cephalosporins: 22 Carbapenems: 8 Macrolides: 4 Penicillin: 33 Linezolid: 6 Polymyxin: 1 Teicoplanin: 1	HT: 75 DM: 45 CVD: 22 CLD: 11 CKD: 1 Cerebrovascular disease: 11 COPD: 10 Cancer: 5 Rheumatic disease: 2
China^20^	350	177 (50.6)	173 Male 177 Female	43 (median)	Moxifloxacin: 156 Levofloxacin: 25 Piperacillin/tazobactam: 9 Unspecified antibiotics: 11	HT: 51 DM: 26 CVD: 15 Chronic pulmonary disease: 7 CKD: 9 CLD: 14 Cancer: 1
China^21^	334	167 (50)	173 female 161 Male	60 (21–90)	Unspecified antibiotics	Not reported
USA^22^	321	222 (69)	155 Male 166 Female	60 ± 17	Unspecified antibiotics	Not reported
USA^23^	242	162 (67)	123 Male 119 Female	50–82	Unspecified antibiotics	COPD: 30 Asthma: 18 Heart failure: 35 Atrial fibrillation: 24 Liver cirrhosis: 8 DM: 118 CKD: 42 Renal disease: 19 Coronary artery disease: 45 HT: 180
China^24^	204	141 (69.12)	107 Male 97 Female	52.91 ± 15.98	Antibiotic treatment	Not reported
China^25^	200	141 (70.5)	98 Male 102 Female	55 ± 17.1	Moxifloxacin, ceftriaxone	Not reported
China^26^	195	115 (59.0)	100 Male 95 Female	64 (median)	Unspecified antibiotics	Not reported
Brazil^27^	181	148 (81.8)	Male 71 110 Female	55.3 ± 21.1	Unspecified antibiotics	Cancer: 181 HT: 77 DM: 31 Chronic renal failure: 10 COPD/asthma: 7
China^28^	169	87 (51.5)	86 Male 83 Female	45 (median)	Unspecified antibiotics	HT: 19 DM: 13 COPD: 3 Cancer: 2 CVD and cerebrovascular diseases: 10
USA, Italy, Spain^29^	144	106 (74)	94 Male 50 Female	62 (median)	Unspecified antibiotics	Kidney transplant: 144
Germany^23^	140	121 (86.4)	90 Male 50 Female	63.5 (17–99)	Ampicillin/sulbactam: 56 Piperacillin/tazobactam: 26 Azithromycin: 38 Meropenem: 6 Moxifloxacin: 4 Cephalosporin: 3	HT: 68 (48.6%) DM: 30 (21.4%) Coronary heart disease: 26 (18.6%) Congestive heart failure: 12 (8.6%) COPD: 7 (5.0%) Bronchial asthma: 15 (10.7%) CKD: 16 (11.4%) Cancer: 29 (20.7%) HIV: 5 (3.6%) CLD: 7 (5.0%)
China^**30**^	138	NA	75 Male 63 Female	56 (median)	Moxifloxacin: 89 Ceftriaxone: 34 Azithromycin: 25	Not reported
China^31^	136	NA	66 Male 70 Female	56 (median)	Moxifloxacin: 51 Cefoperazone-sodium/sulbactam-sodium: 88 Imipenem/cilastatin: 4	Not reported
China^32^	135	131 (97)	57 Male 78 Female	53.53 ± 13.22	Moxifloxacin	Not reported
China^33^	135	59 (43.7)	72 Male 63 Female	47 (median)	Unspecified antibiotics	Not reported
China^34^	132	92 (69.6)	74 Male 58 Female	58.8 ± 12.9	Unspecified antibiotics	CVD: 52 Cancer: 7 CKD: 1
China^35^	107	85 (79.4)	57 Male 50 Female	51 (median)	Unspecified antibiotics	Not reported
China^36^	101	99 (98)	48 Male 53 Female	51 (median)	Unspecified antibiotics	Not reported
China^37^	99	70 (71)	67 Male 32 Female	55 · 5 ± 13 · 1 (21–82)	Cephalosporins, quinolones, carbapenems, tigecycline, and linezolid	Not reported
South Korea^38^	98	98 (100)	38 Male 60 Female	55.4 ± 17.1	Unspecified antibiotics	Not reported
China^39^	93	84 (90.3)	54 Male 39 Female	43 ± 17.34	Moxifloxacin: 54 Levofloxacin: 5 Azithromycin: 1 Amoxicillin: 1 Cefepime: 1 Cefperazone-sulbactam: 1 Cefixime: 1 Other: 23	HT: 6 DM: 6 Heart disease: 3 Stroke: 2 Hypothyroidism: 2 COPD or chronic bronchitis: 2
China^40^	90	47 (52)	48 Male 42 Female	64 (median)	Unspecified antibiotics	CVD: 11 HT: 38 DM: 17 COPD: 4 CKD: 1 Cerebrovascular disease: 6 Cancer: 10
China^41^	85	77 (90.6)	62 Male 23 Female	65.8 ± 14.2	Meropenem: 38 Imipenem/cilastatin: 1 Moxifloxacin: 40 Levofloxacin: 4 Linezolid: 18 Vancomycin: 2 Teicoplanin: 2 Tigecycline: 2 Piperacillin/tazobactam: 9 Ceftriaxone sodium: 3 Cefoperazone/sulbactam: 2 Ceftazidime/tazobactam: 2	Not reported
China^21^	82	68 (82.9)	44 Male 38 Female	74 (34–95)	Unspecified broad-spectrum antibiotics	Cardiac disease, injury, and surgery: 82
Brazil^42^	79	60 (76)	43 Male 36 Female	4 (median)	Unspecified antibiotics	Pediatric: 79
China^43^	74	31 (41.89)	37 Male 37 Female	46.14 ± 14.19	Unspecified antibiotics	Not reported
Italy^44^	70	32 (45.7)	41 Male 29 Female	45–74	Azithromycin	Not reported
China^45^	68	24 (35.3)	25 Male 43 Female	44.3 ± 16.4	Moxifloxacin: 21 Cephalosporin: 9 Azithromycin:2 Amoxicillin: 2	Not reported
UK^46^	68	9 (1.3)	32 Male 36 Female	42.5 (0.5–76)	Doxycycline, moxifloxacin	Not reported
France^47^	66	34 (51.5)	15 Male 51 Female	87.7 ± 9.0	Azithromycin and rovamycin	Not reported
China^48^	64	45 (70.3)	20 Male 44 Female	61 (median)	Unspecified antibiotics	HT: 32
Oman^49^	63	NA	53 Male 10 Female	48 ± 16	Ceftriaxone:50 Azithromycin:45 Piperacillin/tazobactam: 49	Not reported
China^26^	63	47 (74.6)	38 Male 25 Female	65 (57–71)	Unspecified broad-spectrum antibiotics	Diabetic: 63
Saudi Arabia^50^	61	61 (100)	54 Male 7 Female	51 (median)	Azithromycin, ceftriaxone, and piperacillin/tazobactam	DM: 24 HT: 13 Hypothyroidism: 1
Spain^51^	60	5 (8.3)	60 Female	NA	Unspecified antibiotics	Pregnant: 60
NA^52^	58	29 (50.0)	NA	>20 years	Levofloxacin, moxifloxacin, meropenem, and cefixime	Not reported
Europe^53^	57	35 (63)	40 Male 17 Female	65 (57–70)	1 or more unspecified antibiotics and azithromycin as COVID-19 treatment	Liver transplant
Brazil^54^	56	33 (58.9)	39 Male 17 Female	6.2 (median)	Unspecified antibiotics	Pediatric: 56
China^55^	55	29 (52.7)	31 Male 24 Female	44 (median)	Unspecified antibiotics	HT: 8 DM: 5 Respiratory diseases: 4 Thyroid disease: 3 CLD: 3 CKD: 1 CVD: 1
China^32^	52	52 (100)	34 Male 18 Female	71.40 + 9.43	Moxifloxacin	Cardiac disease, injury and surgery: 52
China^56^	47	25 (53.19)	21 Male 26 Female	45 (median)	Unspecified antibiotics	HT: 10 DM: 9 Coronary heart disease: 6 COPD: 10
China^57^	44	16 (36.4)	22 Male 22 Female	(1–18) years	Unspecified antibiotics	Pediatric: 44
China^58^	41	41 (100)	30 Male 11 Female	49 (median)	Unspecified antibiotics	Not reported
China^59^	34	29 (85)	14 Male 20 Female	33 (10.00–94.25) months	Azithromycin was given to 9 patients with pneumonia infection	Pediatric: 34
Italy^60^	33	NA	30 Male 3 Female	64 (median)	Carbapenem: 4 Cephalosporin: 7 Macrolide: 18 Penicillin: 23 Unspecified antibiotics: 2	Heart disease: 14 Lung disease: 4 DM: 2 Autoimmune disease or immunodeficiency: 1
NA^61^	32	18 (56.3)	NA	NA	Initial antibiotic therapy: cefuroxime 7 Amoxicillin-clavulanic acid 1 Piperacillin/tazobactam Subsequent antibiotic therapy: 7 Cases treated with cefuroxime, 1 amoxicillin-clavulanic acid, 1 Ceftazidime, 2 vancomycin 2, flucloxacillin 3	Not reported
China^62^	31	6 (19.4)	NA	7 years and 1 month (6 months–17 years)	Unspecified antibiotics	Pediatric: 31
Iran^63^	30	NA	14 Male 16 Female	0–18 years	Ceftriaxone: 17 Azithromycin: 2 Meropenem: 6 Clindamycin: 3 Vancomycin: 6	Pediatric: 30
China^64^	28	23 (82.1)	17 Male 11 Female	65 (median)	Unspecified antibiotics	Cancer: 28
Italy^65^	25	20 (80)	20 Male 5 Female	71.64 ± 10.08	Ceftriaxone and azithromycin	Cancer: 25
China^66^	25	13 (56)	14 Male 11 Female	3 (2–9)	For 2 critical cases: Case 1: cefoperazone/sulbactam Case 2: meropenem, linezolid	Pediatric: 25
China^57^	23	6 (26.1)	10 Male 13 Female	0 day–1 year	Unspecified antibiotics	Neonate and infant: 23
China^67^	20	17 (85.0)	10 Male 10 Female	43.2 ± 14.0	Unspecified antibiotics	Not reported
China^68^	17	13 (76.5)	12 Male 5 Female	88 (median)	Unspecified antibiotics	HT: 9 CVD: 8 CKD: 6 DM: 5 Neurodegenerative diseases 5 COPD: 3 Cancer: 2
China^69^	16	8 (50)	6 Male 10 Female	44.1 (5–70)	Unspecified antibiotics	Not reported
China^70^	15	15 (100)	Female	32 ± 5	Unspecified antibiotics	Pregnant: 15
China^71^	11	11 (100)	5 Male 6 Female	36.6 (2–69)	Ceftriaxone and moxifloxacin initially and changed to cefoperazone sulbactam, linezolid, and polymyxin later	Not reported
China^72^	10	5 (50)	4 Male 6 Female	74 (3–131) months	Unspecified antibiotics	Pediatric: 10
Spain^73^	10	10 (100)	3 Male 7 Female	54 ± 10	Cephalosporin: 7 Carbapenem: 4 Macrolide: 8 Linezolid: 2	HT: 9 DM: 4 Kidney transplant: 10
China^74^	9	4 (44.4)	5 Male 4 Female	42 (14–56)	Moxifloxacin	Not reported
China^75^	9	9 (100)	Female	29.9 (26–40)	Unspecified antibiotics	Pregnant: 9
NA^76^	8	4 (50)	2 Male 6 Female	5 days–12 month	Amoxicillin, cefotaxime and gentamicin	Neonate and infant: 8
UK^76^	8	4 (50)	2 Male 6 Female	5.1 months (5 days–12 months)	Unspecified antibiotics	Not reported
China^77^	6	6 (100)	2 Male 6 Female	3 (1–7)	Unspecified antibiotics	Not reported
Italy^78^	6	6 (100)	5 Male 1 Female	66.5 (50–82)	Unspecified antibiotics	Not reported
Spain^79^	5	5 (100)	3 Female 2 Male	62 (38–86)	All patient received azithromycin and ceftriaxone In addition, case 1: ceftaroline Case 2 and 5: oral cefixime Case 3: levofloxacin	Not reported
China^80^	5	5 (100)	4 Male 1 Female	≥55 years	Unspecified antibiotics	Not reported
China^82^	5	5 (100)	2 Male 3 Female	50.2 (39–66)	Unspecified antibiotics	Not reported
Spain^83^	5	4 (80)	3 Male 2 Transgender	37.8 (29–49)	Case1: — Case 2: meropenem (for 16 days) Case 3: azithromycin (for 5 days) Case 4: azithromycin (for 5 days), cefixime (for 5 days) Case 5: azithromycin (for 5 days), ceftaroline fosamil (for 7 days), co-trimoxazole (for 21 days, followed by secondary prophylaxis)	HIV: 5
China^82^	5	5 (100)	2 Male 3 Female	50.2 (39–66)	Unspecified antibiotics	HT: 2 CVD: 1
China^81^	5	4 (80)	1 Male 4 Female	65.8 (51–79)	Levofloxacin, moxifloxacin, ceftriaxone, piperacillin-tazobactam, and meropenem	Rheumatic diseases: 5
Australia^84^	5	5 (100)	5 Males	63 (46–74)	Unspecified antibiotics	HT: 2 DM: 2 Aortic valve replacement: 1 Asthma: 1
USA^85^	4	2 (50)	2 Male 2 Female	54.3 (38–64)	Azithromycin, also ceftriaxone, was given to one patient	Cardiac disease, injury, and surgery: 4
Italy^86^	4	4 (100)	2 Male 2 Female	61 (48–70)	Case 1: piperacillin/tazobactam and levofloxacin Case 2: meropenem Case 3: iv meropenem Case 4: piperacillin/tazobactam	Lung transplant: 4
NA^87^	3	3 (100)	3 Male	56 (38–74)	Azithromycin	Not reported
China^88^	3	1 (33.3)	3 Male	7.6 (6–9)	Ceftriaxone	Pediatric: 3
Belgium^89^	3	3 (100)	1 Male 2 Female	51.6 (44–64)	Unspecified antibiotics	CVDs: 1
Philippines^90^	2	1 (50)	1 Male 1 Female	44 years 39 years	Vancomycin	None reported
China^91^	2	2 (100)	1 Male 1 Female	40 years 79 years	Unspecified antibiotics	Renal failure: 2
China^92^	2	2 (100)	Male	47–60	Case 1: moxifloxacin, ceftriaxone, and tazobactam Case 2: moxifloxacin	HIV: 2
Italy^93^	2	1 (50)	Male	69–73	Azithromycin	Cancer: 2
NA^94^	2	1 (50)	1 Male 1 Female	59–75	Sulfamethoxazole-trimethoprim-ds	Heart transplant: 2
China^95^	2	2 (100)	2 Male	51–58	Case 1: moxifloxacin, cephalosporin, linezolid, and meropenem Case 2: moxifloxacin	Case 1: allogeneic bone marrow transplantation Case 2: kidney transplantation
USA^94^	2	1 (50)	1 Male 1 Female	59–75	Case1: cefepime Vancomycin Doxycycline sulfamethoxazole-trimethoprim Tobramycin Linezolid	Case 1 and 2: heart transplant DM, HT, CKD
USA^96^	2	1 (50)	2 Male	NA	Case 2: ceftriaxone, piperacillin-tazobactam	Pediatric: 2
USA^97^	2	2 (100)	1 Male 1 Female	55–57	Azithromycin: 2	Case 1: asthma, HT case 2: DM, HT
Iran^98^	2	1 (50)	2 Male	0 months	Unspecified antibiotics	Neonate and infant: 2
USA^99^	2	2 (100)	2 Female	26–77	Ceftriaxone, azithromycin	Not reported
Switzerland^100^	2	2 (100)	Male	59	Levofloxacin: 1 Amoxicillin/clavulanate: 1	HT: 1
Ireland^101^	1	1 (100)	Male	25	Unspecified antibiotics	Not reported
Japan^102^	1	1 (100)	Male	59	Unspecified antibiotics	Not reported
Taiwan^103^	1	1 (100)	Female	55	Ceftriaxone replaced by oral amoxicillin/clavulanate	Not reported
Bhutan^104^	1	1 (100)	Male	76	Ceftriaxone and doxycycline switched to meropenem and vancomycin	Not reported
Colombia^105^	1	1 (100)	Male	34	Unspecified broad-spectrum antibiotics	Not reported
Japan^106^	1	1 (100)	Female	72	Cefepime and clindamycin phosphate	Not reported
NA^107^	1	1 (100)	Male	33	Piperacillin–tazobactam	Not reported
China^108^	1	1 (100)	Male	23	Meropenem and linezolid	DM: 1
Italy^109^	1	1 (100)	Male	56	Piperacillin/tazobactam	Spinal cord injury patient: 1
China^110^	1	1 (100)	Male	50	Moxifloxacin	Renal failure: 1
NA^111^	1	1 (100)	Male	59	Cefepime, piperacillin/tazobactam, linezolid, gentamicin and meropenem and amikacin	Not reported
Italy^112^	1	1 (100)	Female	54	Unspecified broad-spectrum antibiotics	Diaphragmatic rupture and gastric perforation: 1
NA^113^	1	1 (100)	Male	64	Amoxicillin/clavulanic	Cardiac disease, injury, and surgery: 1
NA^114^	1	1 (100)	Male	63	Piperacillin–tazobactam	Cardiac disease, injury, and surgery: 1
NA^115^	1	1 (100)	Male	37	Piperacillin sulbactam	Cardiac disease, injury, and surgery: 1
NA^116^	1	1 (100)	Male	75	Azithromycin with hydroxychloroquine	HIV: 1
NA^117^	1	1 (100)	Female	56	Zosyn and vancomycin	Liver failure: 1
China^118^	1	1 (100)	Female	62	Meropenem and teicoplanin, followed by linezolid and tigecycline	Cancer: 1
NA^119^	1	1 (100)	Male	63	Ceftizoxime sodium+moxifloxacin to ceftizoxime sodium+teicoplanin	Cancer: 1
Iran^120^	1	1 (100)	Male	15 days	Vancomycin and amikacin	Neonate: 1
Morocco^121^	1	1 (100)	Female	17 months	Amoxicilline-acide clavulanique and azithromycin	Infant: 1
China^122^	1	1 (100)	NA	NA	Meropenem and linezolid	Pediatric: 1
Uganda^123^	1	1 (100)	Female	34 years	Unspecified antibiotics	HIV: 1
UK^124^	1	1	Female	22	Ceftriaxone	None reported
Saudi Arabia^125^	1	1 (100)	Male	45	Meropenem and vancomycin	None reported
India^126^	1	1	NA	1 week	Ampicillin, amoxicillin/clavulanate, meropenem, vancomycin	Neonate and infant: 1
India^127^	1	1	Male	60	Unspecified antibiotics	DM, HT, and biclonal gammopathy: 1
USA^128^	1	1 (100)	Male	23	Unspecified antibiotics	Not reported
UK^129^	1	1 (100)	Male	77	Levofloxacin	HT: 1
US^130^	1	1 (100)	Male	20	Unspecified antibiotics	None reported
USA^131^	1	1 (100)	Male	88	Unspecified antibiotics	HT: 1
India^132^	1	1 (100)	Male	60	Meropenem, vancomycin	DM: 1
USA^133^	1	1 (100)	Male	58	Azithromycin, piperacillin/tazobactam	Not reported
China^110^	1	2 (100)	Male	79	Moxifloxacin	End-stage renal disease: 1
Germany^134^	1	1 (100)	Male	46	Ampicillin/sulbactam	HT: 1
USA^135^	1	1 (100)	Male	24	Vancomycin, cefepime, meropenem	DM: 1
Netherlands^136^	1	1 (100)	Male	7	Amoxicillin	Not reported
Singapore^137^	1	1 (100)	Male	77	Unspecified antibiotics	HT, coronary artery disease, and asthma-COPD overlap syndrome: 1
Niger^138^	1	1 (100)	Male	8 months	Ceftriaxone, gentamycin	Neonate and infant
US^139^	1	1 (100)	Male	49	Ceftriaxone, azithromycin	Not reported
Qatar^140^	1	1 (100)	Female	40	Azithromycin, piperacillin/tazobactam, meropenem	Not reported
Belgium^113^	1	1 (100)	Male	64	Amoxicillin/clavulanate	HT and aortic dissection: 1
Italy^141^	1	1 (100)	Female	78	Ceftriaxone, piperacillin/tazobactam, levofloxacin	Not reported
USA^142^	1	1 (100)	Female	13	Ceftriaxone, metronidazole	Pediatric: 1
China^143^	1	1 (100)	Female	65	Moxifloxacin	Not reported
Brazil^144^	1	1 (100)	Male	65	Meropenem, vancomycin	DM, HT, and cancer: 1
China^145^	1	1 (100)	Male	64	Unspecified antibiotics	Cancer: 1
USA^146^	1	1 (100)	Male	78	Cefepime	Not reported
USA^147^	1	1 (100)	Male	51	Ceftriaxone, azithromycin	Diabetes: 1

CKD, chronic kidney disease; CLD, chronic liver disease; COPD, chronic obstructive pulmonary disease; CVD, cardiovascular disease; DM, diabetes mellitus; HIV, human immunodeficiency virus; HT, hypertension.

**FIG. 2.**
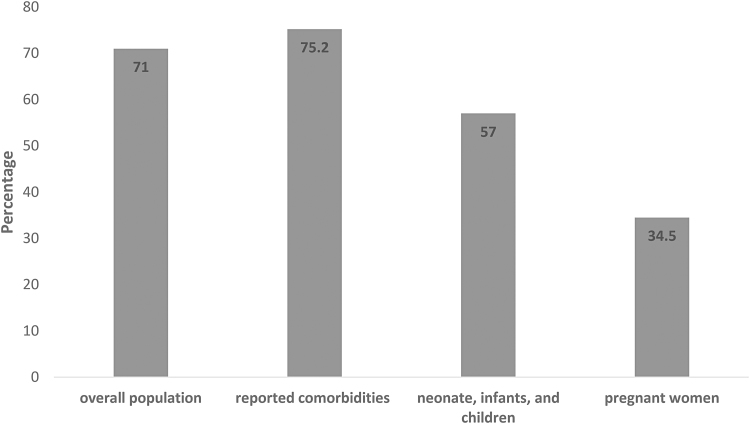
Comparison of percentage antibiotic prescription in studied population compared to patients with reported comorbidities, children, and pregnant women.

The study population's demographic and clinical characteristics included all ages from neonates, children, and adults, including pregnant women and the elderly. Associated underlying conditions included hypertension, diabetes mellitus, heart, respiratory, renal, liver, thyroid, cerebrovascular, rheumatic diseases, and HIV and organ transplantation (heart, lung, kidney, liver, and bone marrow). Of 28,093 patients included in the combined studies, 58.7% received antibiotics (16,490/28,093). The percentage of patients prescribed antibiotics in each article differs, ranging from 1.3% to 100% coverage, with only 9.9% of the articles reporting less than 50% antibiotic covering (14/141). Most included articles did not present clear data on an antibiotic prescription for patients with other complications versus those without comorbidities. Comparing the articles that include the population who suffered from other diseases to those with no other complications, we found that antibiotic coverage did not differ significantly between patients with and without comorbidities (75.2%, 415/552), and 71% (8,449/11,886), respectively (Fig. 2).

Antibiotics coverage was less in children, 57% (187/329) compared to adults, and it was least in pregnant women (34.5%, 29/84). Despite the high percentage of antibiotic prescribing, most articles did not report bacterial coinfection (75.36%), indicating that probably a significant amount of antibiotics were empirically and unnecessarily prescribed.

The spectrum of antimicrobial prescreening is broad since more than 40 different antimicrobials were used to manage patients with COVID-19 disease (Table 2).

**Table 2.** Showing the Number of Articles Reporting Each Antibiotic

Antibiotic	No. of articles
Unspecified antibiotics	68
Cephalosporins	38
Azithromycin	27
Moxifloxacin	23
Meropenem	20
Piperacillin/tazobactam	18
Levofloxacin	13
Linezolid	12
Vancomycin	9
Amoxicillin/clavulanate	8
Teicoplanin	6
Carbapenem	6
Amoxicillin	6
Cefepime	6
Tigecycline	4
Cefoperazone/sulbactam	4
Cefixime	4
Penicillin	4
Doxycycline	4
Fluoroquinolones	3
Imipenem/cilastatin	2
Clindamycin	2
Amikacin	2
Gentamicin	2
Trimoxazole	2
Sulfamethoxazole/trimethoprim	2
Ampicillin/sulbactam	2
Flucloxacillin	1
Ceftazidime/tazobactam	1
Cefotaxime	1
Ceftaroline fosamil	1
Ceftizoxime sodium	1
Meropenem/vancomycin	1
Piperacillin/sulbactam	1
Tazobactam	1
Spiramycin	1
Tobramycin	1
Clarithromycin	1
Ampicillin	1
Tetracycline	1
Polymyxin	1
Metronidazole	1

Inferring from the number of articles reporting the use of specific antibiotics, cephalosporins followed by azithromycin and moxifloxacin were the predominant oral antibiotics while piperacillin/tazobactam was the prevalent parenteral antibiotic. However, when subdividing cephalosporins into distinct classes based on their generation (first vs. second vs. third vs. fourth), azithromycin becomes the predominant antibiotic reported, which reflects its prominent role during the pandemic. Nevertheless, most studies highlighted that the majority of antibiotics were prescribed empirically as prophylaxis to prevent secondary bacterial infection,^70^ to treat secondary bacterial infection such as pneumonia,^59^ or as potential COVID-19 treatment agents.^53^ Other described drugs reported include meropenem, levofloxacin, linezolid, vancomycin, amoxicillin/clavulanate, Teicoplanin, and carbapenem.

## Discussion

The excessive and inappropriate prescribing of antibiotics is a significant challenge for health care across the globe. The escalating problem has been directly associated with detrimental patients' safety through the development of direct adverse events, indirect acquisition of secondary health care-associated infections, propagation of AMR, worsening infection control and prevention measures, as well as substantial cost implications.^148,149^ Of all infectious diseases, respiratory infections are the leading cause of inappropriate antibiotic prescribing and overuse. The majority of upper respiratory tract infections are caused by viruses, and only less than 10% are caused by bacteria^150^; nevertheless, the WHO reported that in 2016, 71% of patients with UTRIs had been prescribed antibiotics.^151^

The COVID-19 pandemic caught all health care settings across the globe by surprise; the novel SARS-CoV-2 virus caused an unprecedented universal health scare since there was little preceding knowledge about the disease and its implications, particularly potential secondary infections. Furthermore, the disease presents primarily as a respiratory illness mimicking bacterial infections hence confounding clinical assessment; conversely, critical patients need invasive procedures often associated with secondary health care-associated infections. To add the disease complexity, unverified early clinical reports and trials advocated using antibiotics to hinder disease progression and hasten viral clearance, despite the discouragement of such an approach by international guidelines.^8^ Consequent to all these factors, antibiotic prescribing was noticeably frequent in patients with COVID-19 disease.

Our search encompassed about 28,000 patients from 28 different countries, to evaluate the problem systematically, the majority of which were severely affected by the pandemic, such as China, Iran, Italy, Spain, UK, and the USA, demonstrated widespread practice of prescribing antibiotics particularly in adults underlying clinical with conditions. The overall percentage of cases prescribed antimicrobial therapy is evident in 58.7% of cases being more common with premorbid or immune-compromised conditions (Fig. 1). Several authors reported treatment strategies for COVID-19 patients incorporating empirical antibiotic treatment.^14,30,37,58,152^ Such observations are in line with early pandemic epidemiological reports since it was apparent that more severe and critical disease is predominant in the elderly and those with underlying premorbid conditions such as diabetes, heart failure, and the immune-compromised. Conversely, severity markers included acute kidney and liver injuries, explaining antibiotic prescribing prevalence in such populations.

It is worth noticing; prescribed antibiotics are not necessarily to cover documented secondary bacterial infections since, in many studies, the presence of bacterial coinfection or secondary infection is much lower than the number of patients prescribed antimicrobial therapy. In their review, Lai *et al.*^153^ reviewed 13 papers for the presence of bacterial coinfection or secondary infection, 5 of which reported 0% bacterial coinfection or secondary infection. In contrast, three reported a low percentage of 1%, 3.4%, and 4.8%, respectively. Similarly, a large-scale study from New York described 5,700 patients with only 3 secondary bacterial infections.^154^ On the contrary, this in contrast with Italy's study, where 17.2% of patients had bacterial pneumonia and 37% suffered from secondary bacteremia.^155^ Lansbury *et al.* covered 30 studies and 3,834 patients, demonstrating only 7% of the hospitalized patients infected with COVID-19 had a bacterial coinfection.^156^ Understandably, the presence of bacterial coinfection was highest in ICU patients (14%) compared to patients in mixed wards (4%). A third review reported 8% of bacterial or fungal coinfection.^7^

The reviewed evidence supports the discrepancy between inappropriate and excessive antibiotic prescribing in patients with COVID-19 disease and the presence of bacterial coinfections. Nevertheless, Chien-Yi Chang and Kok-Gan Chan argue that the low rate of coinfection could result from prescribing antibiotics on a large scale to avoid overwhelming health systems during the early pandemic.^157^ Furthermore, some have argued that the lack of clear antimicrobial stewardship guidance for the frontline clinician at the early stages of the pandemic probably resulted in an inclination toward antimicrobial prescribing, especially in the early stages of the pandemic. In addition, Lansbury *et al.*'s^156^ analysis shows that more than 90% of the patients in 10 out of 17 studies, in which patients were prescribed antibiotics, received the antimicrobial therapy empirically. It is also worth mentioning that in patients with moderate and severe symptoms, those who received antibiotics or corticosteroids had more extended hospital stays than those who did not.^17^

It is worth noting that the high percentage of antibiotic prescribing in patients with no comorbidities (71%) could be confounded by not reporting them in some of the articles, which does not equate to their absence. It is quite possible that an undetermined percentage of patients in such studies suffer from comorbidities. The review also demonstrated lower antibiotic prescribing patterns in the pediatrics population; from 329 neonates, infants, and children included in the review, only 187 (57%) were prescribed antimicrobial therapy. This is a lower rate but might also be appropriate since coinfection is expected in the pediatric population since two studies reported 40% and 51.3% coinfection rates, respectively.^158,159^ This indicates that the pediatric population might have been better managed during the pandemic from the ASP point of view. Pregnant women were the least to be prescribed antimicrobial therapy, with only 34.5%, which might be due to fears of prescribing antimicrobials during pregnancy rather than its liberal use when compared to a similar cohort, however, we are not sure of the reason for this lower rate in antimicrobial prescription in pregnant women.

The macrolide antibiotic azithromycin was the predominant antimicrobial agents reported in the management of COVID-19 disease (Table 2). Most possible, it was used for its claimed anti-inflammatory effect.^160^ Before the start of the pandemic, it was used mostly to treat community-acquired pneumonia as well as exacerbations of chronic obstructive pulmonary disease.^161^ Azithromycin's role has been recognized by previous reports of efficacy against other RNA viruses such as Zika and Ebola virus disease^162–164^ and has been speared when suggested as an adjunct to hydroxychloroquine leading to rapid viral clearance in COVID-19 patients through unclear mechanisms.^9^ This probably reflects the highlighted issue with the drug in the foremost pandemic history.^160^ Although some limited reports support improved outcomes with adjunctive macrolides in the treatment of COVID-19 disease stemming from previous observations of moderate-to-severe acute respiratory distress syndrome, this has not been materialized in COVID-19 clinical trials.^165^ Furthermore, both hydroxychloroquine/chloroquine and azithromycin have been associated with cardiotoxicity by prolonging the QT intervals (the time it takes for the ventricles of the heart to contract and relax), which might precipitate arrhythmias in susceptible patients, particularly those with cardiac diseases, the impact of which is yet to be thoroughly evaluated.^166^ The widely used antibiotic azithromycin was gradually recognized as a rare cause of prolonged QT, severe arrhythmia, and increased risk of sudden death.^167–170^ Beović *et al.*^171^ reported that broad-spectrum antibiotic use in patients with COVID-19 is widespread, according to his survey study administered across 82 hospitals in 23 countries. Importantly, different broad-spectrum antibiotics have been frequently prescribed, including piperacillin/tazobactam, meropenem, vancomycin, and teicoplanin, highlighting potential further development of current or future AMR. More than half of the respondents reported combined use of β-lactams and macrolides or fluoroquinolones, and the most commonly prescribed antibiotic in the COVID-19 ICU was piperacillin/tazobactam.^171^ Worryingly, most broad-spectrum antibiotics have been prescribed empirically as prophylaxis to prevent secondary bacterial infection,^70^ or to treat bacterial secondary infection and pneumonia,^59^ or as part of COVID-19 treatment^53^

Although the systematic search captured a significant number of studies in a short time frame, we acknowledge there are some accompanying limitations. Restricting inclusion to the English language probably omitted other thematic studies. The pandemic's dynamic nature and short time reporting scope probably caused reporting bias, which might be corrected over time. Nevertheless, our report outcomes are in line with other conducted cross-sectional studies such as the WHO studied report.^8^

In summary, this systematic review demonstrated the widespread practice of antibiotic prescribing for COVID-19 patients during the pandemic with little supporting evidence of secondary bacterial infections. While the practice is more frequent in adult patients with comorbidities than in the younger population, this might reflect more advanced and severe diseases in this population. We encourage the appropriate and judicious use of antimicrobials, particularly broad-spectrum antibiotics, to avoid short- and long-term consequences. We anticipate if no appropriate actions have been taken throughout the pandemic through various elements of ASPs or tailored COVID-19 management guidelines, such practice might become an established culture with all its detrimental consequences.
